# Identification and Pyramiding of QTLs for Rice Grain Size Based on Short-Wide Grain CSSL-Z563 and Fine-Mapping of *qGL3–2*

**DOI:** 10.1186/s12284-021-00477-w

**Published:** 2021-04-13

**Authors:** Peixuan Liang, Hui Wang, Qiuli Zhang, Kai Zhou, Miaomiao Li, Ruxiang Li, Siqian Xiang, Ting Zhang, Yinghua Ling, Zhenglin Yang, Guanghua He, Fangming Zhao

**Affiliations:** Rice Research Institute, Academy of Agricultural Sciences, Southwest University, Chongqing, 400715 China

**Keywords:** Rice, Chromosome segment substitution line, Grain size, QTL, Gene pyramid, *qGL3–2*

## Abstract

**Background:**

Chromosome segment substitution lines (CSSLs) can be used to dissect complex traits, from which single-segment substitution lines (SSSLs) containing a target quantitative trait loci (QTL) can be developed, and they are thus important for functional analysis and molecular breeding.

**Results:**

A rice line with short wide grains, CSSL-Z563, was isolated from advanced-generation backcross population (BC_3_F_6_) derived from ‘Xihui 18’ (the recipient parent) and ‘Huhan 3’ (the donor parent). Z563 carried seven segments from ‘Huhan 3’, distributed on chromosomes 3, 7, and 8, with average substitution length of 5.52 Mb. Eleven QTLs for grain size were identified using secondary F_2_ population of ‘Xihui 18’/Z563. The QTLs *qGL3–1*, *qGL3–2*, and *qGL7* control grain length in Z563 and have additive effects to reduce grain length; *qGW3–1* and *qGW3–2* control grain width in Z563 and have additive effects to increase grain width. Four SSSLs, three double-segment substitution lines (D1–D3), and two triple-segment substitution lines (T1 and T2) were developed containing the target QTLs. The genetic stability of eight QTLs, including *qGL3–2*, *qGL3–1*, and *qGL7*, was verified by the SSSLs. D1 (containing *qGL3–2* and *qGL3–1*), D2 (*qGL3–1* and *qGL7*), and T1 (*qGL3–2*, *qGL3–1*, and *qGL7*) had positive epistatic effects on grain length, and their grain length was shorter than that of the corresponding SSSLs. The QTL *qGL3–2* was fine-mapped to a 696 Kb region of chromosome 3 containing five candidate genes that differed between ‘Xihui 18’ and Z563. These results are important for functional research on *qGL3–2* and molecular breeding of hybrid rice cultivars.

**Conclusions:**

The short and wide grain of Z563 was mainly controlled by *qGL3–1*, *qGL3–2*, *qGL7*, *qGW3–1* and *qGW3–2.* The major QTL *qGL3–2* was fine-mapped to a 696 Kb region of chromosome 3 containing five candidate genes. Different QTLs pyramiding displayed various phenotypes. In essence, the performance after pyramiding of genes depended on the comparison between the algebraic sum of the additive and epistatic effects of QTLs in the pyramidal line and the additive effect value of the single QTL. The results lay good foundation in the functional analysis of *qGL3–2* and molecular design breeding of novel hybrid rice cultivars.

## Background

Rice (*Oryza sativa* L.) is the third most widely grown cereal crop in the world, after wheat and maize. With the ongoing decline in arable land area and ever-increasing global population, it has become increasingly important to devise new methods to improve rice yield (Zhao et al. 2016). Rice grain size and shape are complex quantitative traits that include grain length, grain width, and grain length-to-width ratio. These traits affect directly both the yield and the quality of rice (Wang et al. 2020). Numerous genes associated with grain size have been cloned and are involved in regulatory pathways, including endogenous hormone regulation, MAPK signal transduction, transcriptional regulation, G protein signal transduction, and the ubiquitin–proteasome pathway (Li and Li 2016). Genes involved in endogenous hormone regulatory pathways include *OsBZR1*, which is a positive regulator of brassinosteroid signalling (Zhu et al. 2015), and *OsMCA1/PAD*, which regulates gibberellin (GA) metabolism and signal transduction (Liu et al. 2015a). *OsMKK4* encodes a mitogen-activated protein kinase that participates in the MAPK signal transduction pathway (Guo et al. 2018). Genes that participate in transcriptional regulatory pathways include the ERF family transcription factor *OsLG3* (Yu et al. 2017), the GRF-interacting factor *OSMKB3*/*OsGIF1* (Lu et al. 2020), the transcriptional activator *AFG1* (Yu et al. 2020), and *OSWRKY36*, which binds to the *SLR1* promoter and enhances its transcription (Lan et al. 2020). The *GS3* gene plays a key role in the G protein signal transduction pathway and binds competitively to the G protein β subunit of *DEP1* or *GGC2* (Sun et al. 2018). Genes in the ubiquitin–proteasome pathway include *GW2*, which encodes a cyclic E3 ubiquitin ligase (Song et al. 2007), and *OsUBP15*, which encodes an ubiquitin-specific protease (Shi et al. 2019). Many other yield-related genes participate in other pathways, such as *OsACS6*, which encodes a protein homologous to aminotransferase (Matsushima et al. 2016), and *OsACOT*, which encodes acyl-CoA thiesterase (Zhao et al. 2019). Among the aforementioned genes, some regulate positively regulate rice grain size, such as *OsBZR1* (Zhu et al. 2015), *OsMCA1*/*PAD* (Liu et al. 2015a), *OsMKK4* (Guo et al. 2018), *OsLG3* (Yu et al. 2017), *OsMKB3*/*OsGIF1* (Lu et al. 2020), *AFG1* (Yu et al. 2020), *OsACOT* (Zhao et al. 2019), and *OsUBP15* (Shi et al. 2019). Other genes negatively regulate grain size, such as *OsWRKY36* (Lan et al. 2020), *GS3* (Sun et al. 2018), *GW2* (Song et al. 2007), and *OsACS6* (Matsushima et al. 2016). Although numerous genes have been identified in rice, compared with the phenotypic diversity of the grain and the complex underlying molecular mechanisms, identification of additional genes associated with grain size is necessary to satisfy growing calls to improve grain quality in rice breeding.

Chromosome segment substitution lines (CSSLs) contribute to genetic variation and are ideal materials for the identification of quantitative trait loci (QTLs) and multiple-character breeding through gene pyramiding (Balakrishnan et al. 2019). A CSSL development program requires population-wide backcrossing and genome-wide marker-assisted selection (MAS) in combination with selfing. Each CSSL carries a small number of specific marker-defined chromosome segments from the donor parent in a genomic background otherwise identical to that of the recipient parent. Ideally, when each CSSL harbors a single substitution segment from the donor, it can be termed a single-segment substitution line (SSSL) (Zhang et al. 2004; Balakrishnan et al. 2019). In particular, a CSSL is a valuable tool in breeding to broaden the existing genetic pool of a cultivated species and to utilize genetic diversity from wild or distantly related species to overcome reproductive isolation (Balakrishnan et al. 2019; Zhang et al. 2020b). A restorer line is important for utilization of heterosis in rice. ‘Xihui 18’ is an excellent *indica* rice restorer line bred by the Rice Research Institute of Southwest University, China. Its desirable traits include its high combining ability, large panicles, multiple grains, and long slender grains. ‘Huhan 3’ is a *japonica* rice cultivar that shows favorable stress resistance and produces short, broad grains. In this study, we report on the development of a novel rice CSSL with short, broad grains, designated Z563, derived from Xihui 18 as the recipient and Huhan 3 as the donor parent. We mapped QTLs for grain size and developed secondary SSSLs, double-segment substitution lines (DSSLs), and triple-segment substitution lines (TSSLs) for target QTLs, and analysed the effects of pyramiding these QTLs. We fine-mapped the selected QTL *qGL3–2* and analysed candidate genes for the locus.

## Results

### Identification of Substitution Segments in Z563

**Fig. 1:**
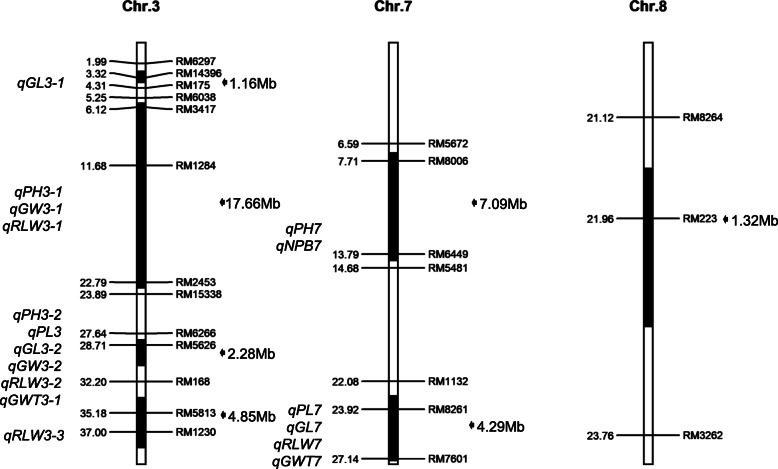
Chromosome substitution segments of Z563 and QTLs identified in the segments. The genome of *indica* rice ‘9311’ was used as the reference genome. Physical distances (Mb) and mapped QTLs are marked on the left of each chromosome. Markers and length of the substitution segments are displayed on the right. The black section of each chromosome represents the substitution segments. GL, grain length; GW, grain width; RLW, grain length-to-width ratio; GWT, 1000-grain weight; PH, plant height; PL, panicle length; NPB, number of primary branches

Following the previous development of Z563, the substitution segments and purity of the genetic backgrounds were investigated with 10 plants of Z563 using 13 markers on the substitution segment and 24 markers outside the substitution segment. All substitution segments of the 10 Z563 plants were identical and no other residual segments derived from Huhan 3 were detected. Z563 harbored seven substitution segments from Huhan 3, which were distributed on chromosomes 3, 7, and 8. The total length of the substitution segment was 38.65 Mb, the maximum length was 17.66 Mb, the minimum length was 1.16 Mb, and the average substitution length was 5.52 Mb (Fig. 1).

### Grain Size and Associated Traits of Z563

**Fig. 2:**
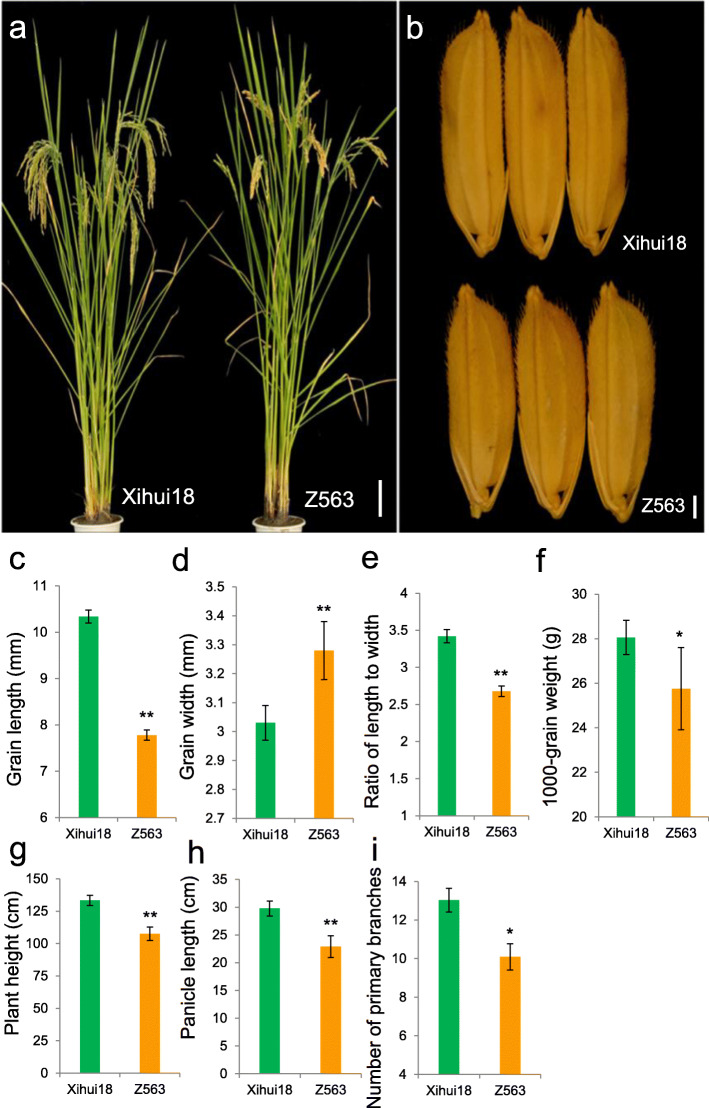
Phenotypic traits of rice ‘Xihui 18’ and Z563. **a** Plant habit. **b** grain size. **c** grain length. **d** grain width. **e** grain length-to-width ratio. **f** 1000-grain weight. **g** plant height. **h** panicle length. **i** number of primary branches. Bars in **a** 10 cm, **b** 1 mm

The plant type of Z563 was similar with that of Xihui 18 (Fig. 2a). The grain width of Z563 differed significantly from that of Xihui 18 (3.03 mm), representing an increase of 0.25 mm (Fig. 2b, d). The grain length, length-to-width ratio, and 1000-grain weight were reduced significantly from those of Xihui 18 (10.34 mm, 3.42, and 28.06 g, respectively), representing decreases of 2.56 mm, 0.74, and 2.30 g, respectively (Fig. 2b, c, e, f).

In addition, in Z563, the associate traits with grain size, such as plant height, panicle length and the number of primary branches, were all decreased significantly than those of Xihui 18, decreasing in turn of 25.74 cm, 6.88 cm and 2.94 primary branches, respectively (Fig. 2g, h, i).

### QTL Mapping for Grain Size and Associated Traits Carried by Z563 in 2018

Z563 carried 11 QTLs for grain size traits, which were located on the substitution segments of chromosomes 3 and 7, respectively (Table 1). Grain length of Z563 was controlled by three major QTLs. The additive effects of *qGL3–1*, *qGL3–2*, and *qGL7* from Huhan 3 decreased the grain length by 0.17 mm, 0.32 mm, and 0.20 mm, respectively, and explained 23.05%, 57.69%, and 25.22% of the phenotypic variation. Grain width of Z563 was controlled by two major QTLs. The additive effects of *qGW3–1* and *qGW3–2* from Huhan 3 increased the grain width by 0.05 mm and 0.03 mm, respectively, and explained 18.46% and 7.98% of the phenotypic variation. Grain length-to-width ratio was controlled by three major QTLs and one minor QTL. The additive effects of *qRLW3–1*, *qRLW3–2* and *qRLW7* from Huhan 3 increased the length-to-width ratio by 0.05 and decreased the ratio by 0.13 and 0.07, respectively, and explained 10.13%, 53.67%, and 19.39% of the phenotypic variation. The additive effect of *qRLW3–3* from Huhan 3 decreased the length-to-width ratio by 0.04 and explained 5.26% of the phenotypic variation. The 1000-grain weight was controlled by one major QTL and one minor QTL. The additive effect of *qGWT3–1* from Huhan 3 decreased 1000-grain weight by 0.92 g and explained 19.83% of the phenotypic variation. The additive effect of *qGWT7* from Huhan 3 decreased the 1000-grain weight by 0.50 g and explained 6.60% of the phenotypic variation (Table 1).

**Table 1:** QTLs for grain-size traits and other associated traits identified in Z563 (2018)

Trait	QTL	Chr.	Linked marker	Additive effect	Variance (%)	*P*-value
Plant height (cm)	*qPH3–1*	3	RM1284	−4.67	36.96	0.0128
	*qPH3–2*	3	RM5626	−1.70	3.64	0.0197
	*qPH7*	7	RM6449	−6.47	59.80	< 0.0001
Panicle length (cm)	*qPL3*	3	RM5626	−0.66	6.64	0.0012
	*qPL7*	7	RM8261	−0.59	5.87	0.0043
Number of primary branch	*qNPB7*	7	RM6449	−0.48	17.79	0.0031
Grain length (mm)	*qGL3–1*	3	RM14396	−0.17	23.05	0.0200
	*qGL3–2*	3	RM5626	−0.32	57.69	< 0.0001
	*qGL7*	7	RM8261	−0.20	25.22	< 0.0001
Grain width (mm)	*qGW3–1*	3	RM1284	0.05	18.46	0.0238
	*qGW3–2*	3	RM5626	0.03	7.98	0.0005
Grain length-to-width ratio	*qRLW3–1*	3	RM3417	0.05	10.13	0.032
	*qRLW3–2*	3	RM5626	−0.13	53.67	< 0.0001
	*qRLW3–3*	3	RM5813	−0.04	5.26	0.0475
	*qRLW7*	7	RM8261	−0.07	19.39	< 0.0001
1000-grain weight (g)	*qGWT3–1*	3	RM5626	−0.92	19.83	< 0.0001
	*qGWT7*	7	RM8261	−0.50	6.60	0.0033

In addition, these grain size QTLs also affected other agronomic traits, such as plant height (PH), panicle length (PL) and the number of primary branches (NPB). For example, *qGW3–1* for grain width and *qPH3–1* for plant height were all linked with the same marker RM1284. While the additive effect of *qGW3–1* increased grain width and *qPH3–1* decreased plant height (Table 1). *qPH3–2* for plant height had the same linkage marker RM5626 with *qPL3*, *qGL3–2*, *qGW3–2*, *qRLW3–2*, *qGWT3–1*. Among them, 5 QTLs except *qGW3–2* were all had additive effects of decreasing values of the according traits. *qPH7* and *qNPB7* were all linked with the same marker RM6449. They all had negative additive effects. *qPL7* shared the same linkage marker RM8261 with *qGL7*, *qRLW7* and *qGWT7*, whose additive effects all decreased values of the according traits (Table 1).

Since some QTLs for associated traits were detected in cluster. Whether are these traits correlated? We conducted analysis of Pearson correlation coefficient for these traits using 184 F_2_ individuals by IBM SPSS Statistics 26. Intrigually, plant height, panicle length, grain length, length-to-width ratio and 1000-grain weight all displayed significant positive correlations each other. Grain width displayed significant negative correlation with plant height (r = − 0.642**), panicle length (r = − 0.648**), the number of primary branches (r = − 0.646**), grain length (r = − 0.777**) and length-to-width ratio (r = − 0.892**). While there was no significant correlation between grain width and 1000-grain weight (Table 2). These results were consistent with the additive effects of QTLs for these traits. Thus, These QTLs for associated traits are pleiotropic.

**Table 2:** Pearson correlation coefficient among grain-size traits and the other associated traits in the F_2_ population

	PH	PL	NPB	GL	GW	RLW	GWT
PH	1						
PL	0.825**	1					
NPB	0.829**	0.844**	1				
GL	0.784**	0.796**	0.797**	1			
GW	−0.642**	−0.648**	−0.646**	−0.777**	1		
RLW	0.773**	0.792**	0.784**	0.977**	−0.892**	1	
GWT	0.345**	0.344**	0.252**	0.503**	−0.082	0.392**	1

** indicate coefficient of correlation between two traits existing significant difference at *p* = 0.01 level, no * indicate no significant difference at *p* = 0.05 level

### Verification and Pyramiding of QTLs Using the SSSLs, DSSLs, and TSSLs in 2020

**Fig. 3:**
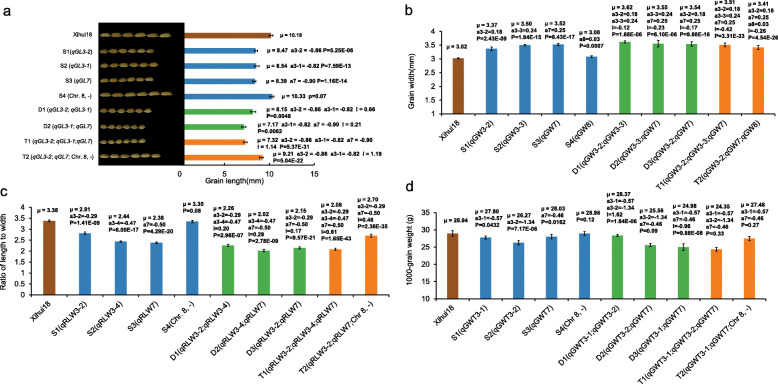
Additive and epistatic effects of grain-size QTLs in single-segment (SSSLs), double-segment (DSSLs), and triple-segment (TSSLs) chromosomal substitution lines (2020). μ is the phenotypic value, a denotes the additive effect of QTLs, I denotes the additive × additive epistatic effect between QTLs. The *P*-value for a SSSL indicates the probability of a significant difference between the SSSL and ‘Xihui 18’, and the SSSL carried a QTL (Student’s *t*-test, *p* < 0.05). The *P*-value for a DSSL and TSSL indicates the probability of an epitatic effect between QTLs in the DSSL or TSSL, i.e., (Xihui 18 + DSSL_*ij*_) and (SSSL_*i*_ and SSSL_*j*_), and (Xihui 18 and Xihui 18 + TSSL_*ijk*_) and (SSSL_*i*_ and SSSL_*j*_ + SSSL_*k*_) (Student’s *t*-test, *p* < 0.05). Chr, Chromosome; S, SSSL; D, DSSL; T, TSSL. S1: Chr3, RM6266--RM5626--RM168; S2: Chr3, RM6297--RM14396--RM175; S3: Chr7, RM1132--RM8261-RM7601--RM22155; S4: Chr8, RM8264--RM223--RM284; D1: Chr3, RM6266--RM5626--RM168, Chr3,RM6297--RM14396--RM175; D2: Chr3, RM6297--RM14396--RM175, Chr7, RM1132--RM8261-RM7601--RM22155; D3: Chr3, RM6266--RM5626--RM168, Chr7, RM1132--RM8261- RM7601--RM22155; T1: Chr3, RM6266--RM5626--RM168, Chr3, RM6297--RM14396--RM175, Chr7, RM1132--RM8261-RM7601--RM22155. T2: Chr3, RM6266--RM5626--RM168, Chr7, RM1132--RM8261- RM7601--RM22155, Chr8, RM8264--RM223--RM284. Internal markers connected with a hyphen indicate the substitution segment from the donor, whereas markers at each end of the substitution segment linked with ‘--’ indicates that segment recombination might occur

Based on the QTL mapping, four SSSLs (S1, S2, S3, and S4), three DSSLs (D1, D2, and D3), and two TSSLs (T1 and T2) were developed in the F_3_ population by MAS (Fig. 3). Eight QTLs (*qGL3–2*, *qGL3–1*, *qGL7*, *qGW3–2*, *qRLW3–2*, *qRLW7*, *qGWT3–1*, and *qGWT7)* were verified in four corresponding SSSLs (S1 to S4), which indicates that the QTLs are genetically stable (Fig. 3a-d). In addition, five QTLs (*qGW3–3*, *qGW7*, *qGW8*, *qRLW3–4*, and *qGWT3–2)* were detected in S2, S3, and S4 (Fig. 3b-d), but were not detected in the secondary F_2_ segregating population of Xihui 18/Z563 (Table 1), which suggests that the SSSLs showed a higher efficiency of QTL detection. *qGW3–1*, *qRLW3–1*, and *qRLW3–3* could not be verified because none of the corresponding SSSLs were developed.

Pyramiding of QTLs for grain size indicated differences in the epistatic effect of different QTLs for the same trait. For example, in D1, pyramiding of *qGL3–2* (additive effect of − 0.86) and *qGL3–1* (additive effect of − 0.82) produced an epistatic effect of 0.66, which reduced grain length of D1 genetically by 1.02 mm (− 0.86–0.82 + 0.66), because the algebraic sum of the additive and epistatic effects (− 1.02) < − 0.86 < − 0.82, resulting in shorter grains (8.15 mm) for D1 than those (8.47 and 8.54 mm) of S1 (with *qGL3–2*) and S2 (with *qGL3–1*) (Fig. 3a). Pyramiding of *qGW3–2* (0.18) and *qGW3–3* (0.24) produced an epistatic effect of − 0.12, which increased grain width of D1 genetically by 0.30 mm, because 0.30 > 0.24 > 0.18, resulting in a broader grain (3.62 mm) for D1 than those (3.37 and 3.50 mm) of S1 (with *qGW3–1*) and S2 (with *qGW3–3*) (Fig. 3b). Pyramiding of *qGWT3–1* (− 0.57) and *qGWT3–2* (− 1.34) produced an epistatic effect of 1.62, which reduced 1000-grain weight of D1 genetically by 0.29 g, because − 0.29 > − 0.57 > − 1.34, resulting in a higher grain weight (28.37 g) for D1 than those (27.80 and 26.27 g) of S1 (with *qGWT3–1*) and S2 (with *qGWT3–2*) (Fig. 3d). These results indicate that pyramiding of these six QTLs produced shorter, broader, and heavier grains than the SSSLs carrying the corresponding single QTL (Fig. 3a, b, d).

In D2, pyramiding of *qGL3–1* (− 0.82) and *qGL7* (− 0.90) produced an epistatic effect of 0.21, which reduced grain length of D2 genetically by 1.51 mm, because − 1.51 < − 0.90 < − 0.82, resulting in shorter grains (7.17 mm) for D2 than those (8.54 and 8.39 mm) of S2 (with *qGL3–1*) and S3 (with *qGL7*) (Fig. 3a). The pyramiding of *qGW3–3* (0.24) and *qGW7* (0.25) produced an epistatic effect of − 0.23, which increased grain width genetically by 0.26 mm, because 0.26 > 0.25 > 0.24, resulting in broader grains (3.55 mm) for D2 than those (3.50 and 3.52 mm) of S2 (with *qGW3–3*) and S3 (with *qGW7*) (Fig. 3b). These results show that pyramiding of *qGL3–1*, *qGL7*, *qGW3–3*, and *qGW7* produced shorter and broader grains than the SSSLs carrying the corresponding single QTL (Fig. 3a, b).

In D3, pyramiding of *qGW3–2* (0.18) and *qGW7* (0.25) produced an epistatic effect of − 0.17, which increased grain width genetically by 0.26 mm, because 0.26 > 0.25 > 0.18, resulting in broader grains (3.54 mm) for D3 than those (3.37 and 3.52 mm) of S1 (with *qGW3–2*) and S3 (with *qGW7*) (Fig. 3b). Pyramiding of *qGWT3–1* (− 0.57) and *qGWT7* (− 0.46) produced an epistatic effect of − 0.96, which reduced the 1000-grain weight genetically by 1.99 g, because − 1.99 < − 0.57 < − 0.46, resulting in a lighter 1000-grain weight (24.98 g) for D3 than those (27.80 and 28.03 g) of S1 (with *qGWT3–1*) and S3 (with *qGWT7*) (Fig. 3d). These results suggest that pyramiding of *qGW3–2*, *qGW7*, *qGWT3–1*, and *qGWT7* resulted in broader but lighter grains than the SSSLs carrying the corresponding single QTL (Fig. 3b, d).

Similarly, in T1, pyramiding of *qGL3–2* (− 0.86), *qGL3–1* (− 0.82), and *qGL7* (− 0.90) produced an epistatic effect of 1.14, which reduced the grain length genetically by 1.44 mm, because − 1.44 < − 0.90 < − 0.86 < − 0.82, resulting in shorter grains (7.32 mm) for T1 than those (8.47, 8.54 and 8.39 mm) of S1, S2, and S3 (Fig. 3a). Pyramiding of *qGW3–2* (0.18), *qGW3–3* (0.24), and *qGW7* (0.25) produced an epistatic effect of − 0.42, which increased the grain width genetically by 0.25 mm, as 0.25 = 0.25 > 0.24 > 0.18, resulting in broader grains (3.51 mm) for T1 than that (3.37 mm) of S1, but basically the same width as those (3.50 and 3.52 mm) of S2 and S3 (Fig. 3b). In T2, pyramiding of *qGL3–2* (− 0.86), *qGL7* (− 0.90), and the substitution segment of the no-grain-length QTL produced an epistatic effect of 1.19, which reduced the grain length by 0.57 mm, because − 0.57 > − 0.86 > − 0.90, resulting in longer grains (9.21 mm) for T2 than those (8.47 and 8.39 mm) of S1 (*qGL3–2*) and S3 (*qGL7*). Pyramiding of *qGW3–2* (0.18), *qGW7* (0.25), and *qGW8* (0.03) produced an epistatic effect of − 0.26, which increased the grain width of T2 by 0.20 mm, because 0.25 > 0.20 > 0.18 > 0.03, resulting in a grain width (3.41 mm) of T2 intermediate between S1 (3.37 mm) and S3 (3.52 mm) (Fig. 3b).

### Fine-Mapping and Candidate Gene Analysis of *qGL3–2*

**Fig. 4:**
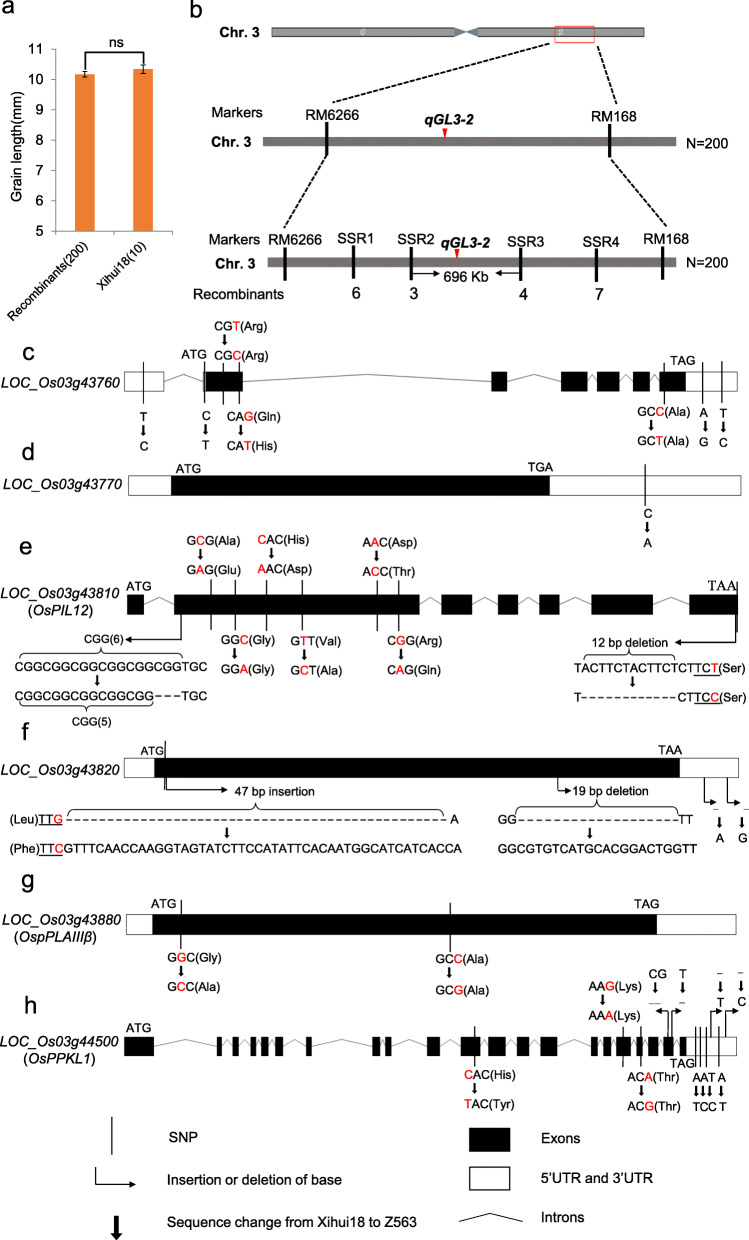
Sequence variation between ‘Xihui 18’ and Z563 in the fine-mapping region for the QTL *qGL3–2*. **a** Grain length of 200 recessive plants (exhibiting a long-grain phenotype) in the F_3_ population and 10 individuals of Xihui 18. **b** Fine-mapping of *qGL3–2*. **c–h** Sequence variation between Xihui 18 and Z563 at the loci **c** *LOC_Os03g43760*, **d** *LOC_Os03g43770*, **e** *LOC_Os03g43810* (*OsPIL12)*, **f** *LOC_Os03g43820*, **g** *LOC_Os03g43880* (*OspPLAIIIβ)*, **h** *LOC_Os03g44500* (*OsPPKL1*)

In order to fine-map *qGL3–2*, 34 simple sequence repeat (SSR) markers between the RM6266 and RM168 were designed. Four SSR markers that displayed polymorphism were used to fine-map *qGL3–2* using 200 recessive plants (long grain phenotype) in the F_3_ population. The mean grain length of the 200 recessive plants was 10.17 mm, which was not significantly different from that of Xihui 18 (10.34 mm) (Fig. 4a). *qGL3–2* was delimited within a region of 696 Kb between SSR2 and SSR3 (Fig. 4b).

Seven candidate genes associated with grain size were predicted, namely *LOC_Os03g43760*, *LOC_Os03g43770*, *LOC_Os03g43810* (*OsPIL12*), *LOC_Os03g43820*, *LOC_Os03g43880* (*OspPLAIIIβ*), *LOC_Os03g43890* (*OsMSI1*), and *LOC_Os03g44500* (*OsPPKL1*) (Fig. 4c-h). We sequenced these genes in Xihui 18 and Z563. *LOC_Os03g43760*, which encoded a protein kinase, carried two single nucleotide polymorphism (SNP) differences in the 5′ untranslated region (UTR) and 3′ UTR, respectively. In addition, three SNP differences in the coding DNA sequence (CDS) were detected. The 177th base of the CDS was changed from G of Xihui 18 to T of Z563, which resulted in a mutation from Gln of Xihui 18 to His of Z563. The other two SNP differences did not cause amino acid changes (Fig. 4c). *LOC_Os03g43770*, which was a cytokinin-like F-box gene, showed one SNP difference between Xihui 18 and Z563 in the 3′ UTR region. The protein-coding region was unchanged, thus this locus was unlikely to be a candidate gene for *qGL3–2* (Fig. 4d). Compared with Xihui 18, *LOC_Os03g43810* (*OsPIL12*) contained a three-base insertion at the 173rd base of the CDS and a 12-base insertion at the 1478th base of the CDS. In addition, seven SNP differences were detected, of which five caused amino acid mutations and two nonsense mutations (Fig. 4e). *LOC_ Os03g43820* showed one SNP difference from the G of Xihui 18 to the C of Z563 at the 18th base of the CDS, which resulted in a mutation from Leu of Xihui 18 to Phe of Z563. A 47-base sequence was inserted at the 19th base of the CDS, and a 19-base sequence was inserted at the 576th base of the CDS, which resulted in multiple amino acid changes, and two bases were inserted in the 3′ UTR (Fig. 4f). The CDS of *LOC_ Os03g43880* (*OspPLAIIIβ*) showed two SNP differences between Xihui 18 and Z563. The 86th base of the CDS was changed from G of Xihui 18 to C of Z563, which caused an amino acid mutation from Gly to Ala. The other SNP difference did not cause an amino acid change (Fig. 4g). *LOC_Os03g43890* (*OsMSI1*) showed no DNA sequence differences between Xihui 18 and Z563, and therefore was not a candidate gene for *qGL3–2*. Compared with Xihui 18, *LOC_Os03g44500* (*OsPPKL1*) showed four SNP differences and a two-base insertion in the 3′ UTR, and three SNP differences in the CDS. The 1495th base of the CDS was changed from C of Xihui 18 to T of Z563, which caused an amino acid change from His to Tyr. The other two SNP differences did not cause an amino acid change. In addition, a two-base deletion at the 2802nd and a one-base deletion at the 2813rd base of the CDS were detected (Fig. 4h). Therefore, *LOC_Os03g43760*, *LOC_Os03g43810* (*OsPIL12*), *LOC_Os03g43820*, *LOC_Os03g43880* (*OspPLAIIIβ*), and *LOC_Os03g44500* (*OsPPKL1*) may be candidate genes for *qGL3–2*.

## Discussion

### Z563 and its Secondary Substitution Lines Show Potential Application in Breeding of Novel Hybrid Rice Cultivars

The utilization of heterosis is of crucial importance in rice breeding. Hybrid rice that shows strong heterosis bred from restorer lines and sterile lines have greatly improved the yield of rice (Wu et al. 2018). Therefore, superior restorer lines are important for breeding hybrid rice cultivars with high yield and good quality. Xihui 18, an *indica* restorer line bred by Southwest University, shows the characteristics of high combining ability, good flowering habit, and long and slender grains. In this study, Xihui 18 was used as the recipient parent to develop a rice line (CSSL-Z563) that harbors seven substitution segments and produces short, broad grains, as well as nine secondary substitution lines. Compared with Xihui 18, these substitution lines contained one-to-seven substitution segments derived from Huhan 3, which changed the long slender grains of Xihui 18 into the short broad grains of the substitution lines, but otherwise their genetic backgrounds were consistent with Xihui 18. In addition, the four fertility restoration genes *Rf-1* (Akagi et al. 2004), *Rf2* (Itabashi et al. 2011), *Rf3* (Cai et al. 2013), and *Rf4* (Kazama and Toriyama 2014) were not substituted. Therefore, Z563 and its secondary substitution lines show potential for use as novel restorer lines and to be crossed directly with male-sterile lines to breed new hybrid cultivars. These lines thus have important potential applications in hybrid rice breeding.

### Comparison of the QTLs Identified in this Study with Previously Reported Genes

A total of 11 QTLs for grain size were identified in the secondary F_2_ population of Xihui 18/Z563. *qGL3–1* was mapped in RM6297--RM14396--RM175, and *OsMCA1/PAD* was located in this substitution interval. *OsMCA1* is homologous to Arabidopsis mechanosensitive calcium channels, and the *pad* mutant produces significantly shorter grains and reduced 1000-grain weight (Liu et al. 2015a), and thus may be a candidate gene for *qGL3–1*. *qGL7*, *qRLW7* and *qGWT7* were mapped in RM1132--RM8261-RM7601--RM22155, and *GL7*, *GL7NR* and *GE* were located in this substitution interval. *GL7* is a major QTL controlling grain length and width. Overexpression of *GL7-S1* or *GL7-S2* increases the ratio of length to width of rice (Wang et al. 2015). *GL7NR* may be a negative regulator of *GL7*. *GE* encodes the CYP78A13 protein (Wang et al. 2015). CYP78A13 activation promotes cell proliferation and has the potential to increase plant height and improve seed yield (Xu et al. 2015). The *bg2-D* mutant was higher compared to the wild type, while grain length, grain width, grain thickness, and 1000-grain weight were significantly increased (Xu et al. 2015). *GE*, *GL7* and *GL7NR* may be candidate genes for *qGL7*, *qRLW7* and *qGWT7*. The question of whether or not these genes are associated with the QTL alleles requires further sequencing and functional complementary verification.

Given that *qGL3–2* contributed 57.69% to phenotypic variation for grain length, substantially higher than *qGL3–1* (23.05%) and *qGL7* (25.22%) in 2018, we further fine-mapped *qGL3–2* to a 696 Kb interval. Seven genes potentially associated with grain length development were identified in this interval. Sequencing revealed that two of these genes showed no differences or did not cause amino acid changes between Xihui 18 and Z563. In contrast, many sequence differences between Z563 and Xihui 18 that led to amino acid variation were detected in *LOC_Os03g43760* (which encodes a protein kinase domain-containing protein), *LOC_Os03g43810* (*OsPIL12*), *LOC_Os03g43820* (plant invertase/pectin methylesterase inhibitor domain-containing protein), *LOC_Os03g43880* (*OspPLAIIIβ*), and *LOC_Os03g44500* (*OsPPKL1/GL3.1*). These genes are potential candidate genes for *qGL3–2*. Among the genes, *LOC_Os03g43760*, *LOC_Os03g43820*, *LOC_Os03g43810* (*OsPIL12*), and *LOC_Os03g43880* (*OspPLAIIIβ*) have not been cloned. The *Arabidopsis thaliana* genome contains a small subfamily of plant pigment-interacting basic helix-loop-helix (bHLH) factors, which are collectively termed PHYTOCHROME INTERACTING FACTOR-LIKE (PIL) family proteins. *LOC_Os03g43810* (*OsPIL12*), a highly homologous member of this family in rice, functions to interact with the *OsPRR1* rhythm element; in addition, the expression of *OsPIL13* is controlled by circadian rhythms (Nakamura et al. 2007). *OspPLAIIIβ* and *pPLAIIIα* both belong to the patatin-related phospholipase A (pPLA) family. The pPLA family members hydrolyze glycerolipids to produce fatty acids and lysophospholipids. *pPLAIIIα* plays an important role in vegetative and reproductive growth of rice, and high activity of pPLAIIIα inhibits cell elongation (Liu et al. 2015b). The *LOC_Os03g43880* gene encodes a pPLAIIIβ protein. However, its function in rice remains unknown. *OsPPKL1*/*GL3.1* encodes a protein phosphatase kelch (PPKL) family–Ser/Thr phosphatase (Zhang et al. 2012). *GL3.1* controls rice seed size and yield by direct dephosphorylation of the substrate cyclin-T1;3, and down-regulation of *GL3.1* in rice results in shorter grains (Qi et al. 2012). *qGL3–2* may represent a novel allele of *OsPPKL1*. *qGL3–2* differed from the mutation site of *GL3.1* reported in previous studies. The allelic mutation of *qGL3–2* from Huhan 3 changed the long grain of Xihui 18 to a short-grain phenotype. Wang et al. (2020) showed that the *qKL3* allele of *OsPPKL1* from Xihui 18 altered the grain phenotype of Z741 (which has a ‘Nipponbare’ background) to a long grain. These results imply that allelic variation of the same gene is an important cause of phenotypic genetic diversity. *LOC_Os03g43760* and *LOC_Os03g43820* have not been identified. Functional complementation of these genes is in progress to determine their contribution to the reduced grain length of Z563.

### SSSLs, DSSLs, and TSSLs Are more Favorable for Analysis of Complex Genetic Characteristics and Molecular Breeding

In this study, based on QTL mapping, the SSSLs for four target QTLs from Z563 were developed and eight QTLs (*qGL3–2*, *qGL3–1*, *qGL7*, *qGW3–2*, *qRLW3–2*, *qRLW7*, *qGWT3–1*, and *qGWT7*) were validated. In addition, S1, S2, S3, and S4 revealed a number of minor QTLs not detected in the secondary F_2_ segregating population of Xihui 18/Z563, such as *qGW3–3*, *qGW7*, *qGW8*, *qRLW3–4*, and *qGWT3–2*, which indicated that a SSSL shows higher sensitivity for QTL detection. Zhao et al. (2016) and Eshed and Zamir (1995) reported that SSSLs show higher QTL detection efficiency and are important for genetic resolution of complex phenotypic traits.

In addition, we analysed the additive and epistatic effects and pyramid performance of *qGL3–2*, *qGL3–1*, and *qGL7* for grain length, *qGW3–2*, *qGW3–3*, *qGW7*, and *qGW8* for grain width, and *qGWT3–1*, *qGWT3–2*, and *qGWT7* for grain weight using three DSSLs (D1, D2, and D3) and two TSSLs (T1 and T2). However, the results obtained varied; for example, pyramiding of short-grain QTLs (*qGL3–2* and *qGL3–1*; *qGL3–1* and *qGL7*) resulted in shorter grains. Pyramiding of broad-grain QTLs (*qGW3–2* and *qGW3–3*; *qGW3–2* and *qGW7*) resulted in broader grains. Pyramiding of QTLs for decreased grain weight resulted in either heavier grains (*qGWT3–1* and *qGWT3–2*) or lighter grains (*qGWT3–1* and *qGWT7*) than the SSSL carrying a single QTL. However, in essence, the performance after pyramiding of genes depended on the comparison between the algebraic sum of the additive and epistatic effects of QTLs in the pyramidal line and the additive effect value of the single QTL. Zhao et al. (2012) argued that whether a larger or smaller value for yield-related traits is produced depends on the difference between the absolute value of the genetic effect (algebraic sum of additive and epistatic effects) in the DSSL and the largest additive effect value in the SSSL. Our results are generally consistent with this finding. However, it can be posited that pyramiding of different QTLs produces different epistatic effects and their performance after pyramiding depends on the comparison between the algebraic sum of additive and epistatic effects of QTLs in the pyramidal line and the additive effect of all single QTLs. On the basis of this rule, we can predict the phenotype of novel pyramided genotypes and select suitable genotypes according to the specific breeding goals, thus realizing the concept of molecular breeding. For example, if a long-grain phenotype is desired from a short-grain line, the selected QTLs should adhere to the condition that the algebraic sum of the additive and epistatic effects of QTLs in the pyramidal line is larger than the maximum additive effect of a single QTL. If the objective is transformation of long grains into short grains, the sum of the additive and epistatic effects of QTLs should be less than the additive effect of any single QTL. If an intermediate type is required, the algebraic sum greater than the minimum additive effect and less than the maximum additive effect of another QTL should be selected. Thus, the present results will be useful in molecular breeding for QTLs with known additive and epistatic effects.

## Conclusions

Using an excellent *indica* restorer line Xihui18 as the genetic background, we identified the rice short and wide grain CSSL Z563. Z563 carried seven substitution segments derived from Huhan3 with an average substitution length of 5.52 Mb. Eleven QTLs were distributed on chromosomes 3 and 7 in Z563. The QTLs *qGL3–1*, *qGL3–2*, and *qGL7* control grain length in Z563 and have additive effects to reduce grain length; *qGW3–1* and *qGW3–2* control grain width in Z563 and have additive effects to increase grain width. Then, four SSSLs, three DSSLs (D1–D3), and two TSSLs (T1 and T2) were developed containing the target QTLs. The genetic stability of eight QTLs, including *qGL3–2*, *qGL3–1*, and *qGL7*, was verified by the SSSLs. D1 (containing *qGL3–2* and *qGL3–1*), D2 (*qGL3–1* and *qGL7*), and T1 (*qGL3–2*, *qGL3–1*, and *qGL7*) had positive epistatic effects on grain length, and their grain length was shorter than that of the corresponding SSSLs. While other QTLs pyramiding displayed different phenotypes. In essence, the performance after pyramiding of genes depended on the comparison between the algebraic sum of the additive and epistatic effects of QTLs in the pyramidal line and the additive effect value of the single QTL. On the basis of this rule, we can predict the phenotype of novel pyramided genotypes and select suitable genotypes according to the specific breeding goals in molecular breeding. Finally, the QTL *qGL3–2* was fine-mapped to a 696 Kb region of chromosome 3 containing five candidate genes that differed between ‘Xihui 18’ and Z563. The results lay good foundation in the functional analysis of *qGL3–2* and molecular design breeding of novel hybrid rice cultivars.

## Materials and Methods

### Development of Z563

A rice CSSL with short, broad grains and designated Z563, was used in this study. Z563 was developed by continuous backcrossing and selfing in combination with MAS between ‘Xihui 18’ (the recipient parent) and ‘Huhan 3’ (the donor parent). First, 241 markers polymorphic between Xihui 18 and Huhan 3 were selected from among 429 markers that covered the entire rice genome. Twenty plants were selected from the BC_2_F_1_ generation and each line of each generation for MAS. In the BC_3_F_6_ generation, a CSSL with short broad grains and harboring seven substitution segments was developed and designated CSSL-Z563. The identification of substitution segments was performed as described previously (Zhao et al. 2016). The estimated length of the substitution segments was calculated following an established method (Paterson et al. 1991). The chromosome substitution segment map was constructed using Mapchart 2.32 software (https://www.wur.nl/en/show/Mapchart.htm).

### Material for QTL Mapping

The QTL mapping population was derived from a secondary F_2_ segregating population consisting of 184 plants raised from the cross between Xihui 18 and Z563.

### Material for Fine-Mapping of *qGL3–2*

Material for fine-mapping of the QTL *qGL3–2* comprised the recessive plants (long grain phenotype) in the F_3_ population developed from four F_2_ recombinant plants in which the *qGL3–2* locus was heterozygous and all other loci were consistent with the receptor parent Xihui 18.

### Materials for SSSLs, DSSLs, and TSSLs

Nine individuals carrying the target QTL and the least heterozygous markers and the most markers same with Xihui 18-type band were selected from the F_2_ population and planted as lines in 2019. Leaves of 20 plants from each line were sampled for DNA extraction, and PCR amplification and polyacrylamide gel electrophoresis were conducted for the development of SSSLs, DSSLs, and TSSLs.

### Material Planting Method

In July 2017, the F_1_ population was generated at the experimental station of Southwest University in Chongqing, China by crossing Xihui 18 with Z563, and the hybrid was harvested. In September, the F_1_ individuals were planted in Linshui, Hainan province and the F_1_ grains were harvested. In March 2018, Xihui 18, Z563, and the F_2_ population were planted at the experimental station of Southwest University. On 13 April, 30 plants of Xihui 18 and Z563 and 184 plants of the F_2_ population were transplanted to the same field. The spacing between the hills and rows was 16.67 cm × 26.67 cm. In 2019, nine plants for secondary substitution development were selected from the F_2_ population, 30 individuals for Xihui 18, and all plants of four recombinant lines selected for fine-mapping of *qGL3–2* were planted and transplanted in the same manner as that of 2018. In 2020, four SSSLs, three DSSLs, and two TSSLs developed from the F_3_ generation and Xihui 18 were planted and transplanted in the same manner with 30 plants for each material. Conventional field management practices were applied.

### Measurement of Grain Size Traits

Grains from 10 plants of Xihui 18, Huhan 3, Z563, SSSLs, DSSLs, and TSSLs, and 184 plants of the F_2_ population were harvested at maturity. The grain length, grain width, length-to-width ratio, and 1000-grain weight were measured following the method of Wang et al. (2020). A Student^’^s *t*-test was conducted for each trait to access the significance of differences between Xihui 18 and Z563. The mean and standard deviation for each trait were calculated using Microsoft Excel 2016.

### Method of QTL Mapping

A total of 184 plants of the secondary F_2_ population were used for QTL mapping. DNA from each sample was extracted using the cetyltrimethyl ammonium bromide method. PCR amplification, polyacrylamide gel electrophoresis, and rapid silver staining were conducted following the methods described by Zhao et al. (2016). The Xihui 18-type band was scored as ‘− 1’, the Z563-type band as ‘1’, the heterozygote as ‘0’, and a missing band as ‘.’. The mean for each trait from 184 F_2_ plants and the marker assignment value were used for QTL mapping. The restricted maximum likelihood method implemented in the HPMIXED program of SAS 9.3 (http://suportsus.com/publishing) was used to plot the QTL. The significance level *P* < 0.05 was used as the threshold to determine whether the QTL was associated with the marker on the substitution segment.

### Method of Verification and Pyramiding of QTLs Using SSSLs, DSSLs, and TSSLs

In 2020, 10 plants of Xihui 18 and each SSSL, DSSL, and TSSL were sampled after maturity. Grain size-related traits were measured, with three replicate measurements recorded per plant. Given that only one substitution segment differed between each SSSL and the recipient parent Xihui 18, under a specific environment (the same year and same experimental field with no replicate plot), the genetic model for Xihui 18 was *P*_0_ = μ + ε, and that for the SSSL carrying a specific QTL was *P*_*i*_ = μ + *a*_*i*_ + ε, where *P*_0_ and *P*_*i*_ represent the phenotype value of any plant in the plot of Xihui 18 and the SSSL_*i*_ carrying the substitution segment *i*, μ represents the mean value for the Xihui 18 population, *a*_*i*_ represents the additive effect of the QTL, and ε represents the random error. Statistical differences between each SSSL and Xihui 18 were analysed using the Student’s *t*-test, and a QTL was considered to exist when the *P*-value was less than 0.05. The additive effect of the QTL was calculated as half the difference between the mean phenotypic values of the SSSL and Xihui 18 (Zhang et al. 2020a). All calculations were conducted using Microsoft Excel 2016.

Under the same environment, the genetic model for DSSLs and TSSLs was *P*_*ij*_ = μ + *a*_*i*_ + *a*_*j*_ + I_*ij*_ + ε and *P*_*ijk*_ = μ + a_*i*_ + a_*j*_ + a_*k*_ + I_*ijk*_ + ε, respectively, where *P*_*ij*_ and *P*_*ijk*_ represent the phenotype value of any plant in the plot of the DSSL_*ij*_ and TSSL_*ijk*_, *a*_*i*_, *a*_*j*_, and *a*_*k*_ represent the additive effect of the QTL in substitution segment *i*, *j*, and *k*, respectively, *I*_*ij*_ and *I*_*ijk*_ represent the *a*_*i*_*a*_*j*_ epistatic effect between QTLs in substitution segment *i* and *j*, and *a*_*i*_*a*_*j*_*a*_*k*_ epistatic effect between QTLs in substitution segment *i*, *j*, and *k*. Thus, the epistatic effect between QTLs in the DSSL was tested for the significance of each trait between (Xihui 18 + DSSL_*ij*_) and (SSSL_*i*_ + SSSL_*j*_) using the Student’s *t*-test, where SSSL_*i*_, SSSL_*j*_, DSSL_*ij*_, and Xihui 18 represent the phenotypic value of a trait corresponding to the SSSL, DSSL, and Xihui 18, respectively. An epistatic effect between QTLs was considered to exist when the *P*-value was less than 0.05. The epistatic effects between non-allelic QTLs were estimated as half the mean phenotypic values of (Xihui 18 + DSSL_*ij*_) − (SSSL_*i*_ + SSSL_*j*_) (Zhang et al. 2020a). For the epistatic effect between QTLs in a TSSL, the significance of each trait between (Xihui 18 + Xihui 18 + TSSL_*ijk*_) and (SSSL_*i*_ + SSSL_*j*_ + SSSL_*k*_) was tested using the Student’s *t*-test, where TSSL_*ijk*_ represents the phenotypic value of the TSSL; an epistatic effect between QTLs was considered to exist when the *P*-value was less than 0.05. The epistatic effect of QTLs in the TSSL was estimated as half the mean phenotypic value of (Xihui 18 + Xihui 18 + TSSL_*ijk*_) − (SSSL_*i*_ + SSSL_*j*_ + SSSL_*k*_). All statistical analyses were conducted using Microsoft Excel 2016.

### Fine-Mapping and Candidate Gene Sequencing of *qGL3–2*

Based on the mapping of *qGL3–2*, new molecular markers were synthesized to analyse the linkage using the recessive plants (long grain phenotype) in the F_3_ population derived from four plants recombinant at the *qGL3–2* locus but with otherwise identical genetic backgrounds to Xihui 18.

All candidate gene information within the fine-mapped interval *of qGL3–2* was predicted and combined with gene annotations to select possible candidate genes with Gramene (http://www.gramene.org/) and the China National Rice Database Center (http:/www.ricedata.cn/). Primers were then designed using Vector NTI software, and the target gene was amplified using Takara’s Primer STAR Max DNA Polymerase with Xihui 18 and Z563 as templates. The PCR products were sequenced by Tsingke Biological Technology Co., Ltd. (Chongqing, China).

## Data Availability

The datasets supporting the conclusions of this article are included within the article.

## Abbreviations

QTL: Quantitative trait loci
CSSLs: Chromosome segment substitution lines
SSSL: Single-segment substitution lines
DSSL: Double-segment substitution lines
TSSL: Triple-segment substitution lines
SSR: Simple sequence repeat
MAS: Marker-assisted selection
GL: Grain length
GW: Grain width
RLW: Grain length-to-width ratio
GWT: 1000-grain weight
PH: Plant height
PL: Panicle length
NPB: Number of primary branches
SNP: Single nucleotide polymorphism
UTR: Untranslated region
CDS: Coding DNA sequence
